# Transforming growth factor serum concentrations in patients with proven non-syndromic aortopathy

**DOI:** 10.3389/fcvm.2022.980103

**Published:** 2022-09-06

**Authors:** Mikita Karalko, Marek Pojar, Lenka Zaloudkova, Vaclav Stejskal, Salifu Timbilla, Pavla Brizova, Jan Vojacek

**Affiliations:** ^1^Department of Cardiac Surgery, Faculty of Medicine, University Hospital Hradec Králové, Charles University, Hradec Králové, Czechia; ^2^Institute of Clinical Biochemistry and Diagnostics, Faculty of Medicine in Hradec Králové, University Hospital Hradec Králové, Charles University, Hradec Králové, Czechia; ^3^The Fingerland Department of Pathology, Faculty of Medicine in Hradec Králové, University Hospital Hradec Králové, Charles University, Hradec Králové, Czechia

**Keywords:** ascending aorta dilatation, transforming growth factor β1, aortopathy, aortic disease, biomarker

## Abstract

**Background:**

The mechanism underlying aortic dilatation is still unknown. Vascular dilatation is thought to be the result of progressive aortic media degeneration caused by defective vascular matrix hemostasis, including TGF-β1 dysregulation. The goal of this study is to draw attention to the potential utility of TGF-β1 as a diagnostic marker in non-syndromic patients with aortic dilatation.

**Methods:**

TGF-β1 levels in plasma were measured in 50 patients who had undergone surgery and had a tricuspid or bicuspid aortic valve as well as a normal or dilated ascending aorta. A pathologist also examined thirty resected aorta samples. To specify the reference range of TGF-β1, a control group of 40 volunteers was enrolled in this study.

**Results:**

We discovered a significant difference in TGF-β1 levels between patients with aortic dilatation and the control group (32.5 vs. 63.92; *P* < 0.001), as well as between patients with non-dilated aorta but with aortic valve disease, and the control group (27.68 vs. 63.92; *P* < 0.001). There was no difference between the dilated ascending aorta group and the non-dilated ascending aorta group. We found a poor correlation between TGF-β1 levels and ascending aorta diameter as well as the grade of ascending aorta histopathological abnormalities.

**Conclusion:**

TGF-β1 concentration does not meet the criteria to be a specific marker of aortic dilatation, but it is sensitive to aortic valvulopathy-aortopathy. A larger patient cohort study is needed to confirm these findings.

## Introduction

Ascending aortic dilatation is the most common pathological aortic condition associated with a risk of dissection or rupture (1). The pathophysiological mechanism leading to aortic dilatation is still poorly understood. It is thought that vessel dilatation is the consequence of a progressive degeneration of the aortic media, caused by defective vascular matrix hemostasis involving TGF-β1 dysregulation (2–5). TGF-β1 is a cytokine that participates in a board range of cellular regulatory processes and is associated with various diseases including aortic aneurysm. It is well-known that increasing levels of TGF-β1 are associated with Marfan syndrome (MFS) caused by FBN1 mutation and subsequent defects in the signaling system (6). TGF-β1 is synthesized as an inactive protein, named latent TGF-β, that consists of a main region and a latency associated peptide (LAP) (7).

The TGF-β1 pathway is increasingly attracting attention, by virtue of its role in fibrosis, inflammation, cell proliferation and migration, extracellular matrix (ECM) remodeling, and in light of its involvement in aortopathy syndromes such as Loeys-Dietz and Marfan syndromes (8).

Although aortic dilatation is more severe and occurs earlier in patients with bicuspid aortic valve (BAV) than with normal, tricuspid aortic valve (TAV), the later may also cause development of aortopathy in elderly patients, with histopathological findings similar to that of BAV associated with aortic dilatation (9).

Aneurysm formation in histological findings is associated with apoptosis of vascular smooth muscle cells (VSMCs). VSMC nuclei loss is a typical finding in an aneurysmatic aorta. It has been proposed that apoptosis of VSMC contributes to the development of aortic dissection (10–12). Vascular smooth muscle cells (VSMCs) are the major cell type in the tunica media of blood vessel wall and key players in the regulation of blood pressure and flow. VSMCs also maintain the matrix components of the media, and their dysfunction results in the remodeling of the aortic wall (13).

The aim of this study is to compare serum TGF-β1 concentrations in patients with a tricuspid or bicuspid aortic valve and a normal or dilated ascending aorta. We also sought to find a correlation between TGF-β1 concentrations and aortic wall diameter and to ascertain histopathological changes in the aortic wall.

## Material and methods

### Patients

The study included 50 patients who underwent surgery at the University Hospital Hradec Králové’s Department of Cardiothoracic Surgery (UHHK). Patients with ascending aorta aneurysm (*n* = 30) or isolated aortic valve stenosis or insufficiency (*n* = 20) were recruited in a series from 2017 to 2020. Table 1 shows the clinical characteristics of the study subjects. The subjects were split into two groups. Dilated ascending aorta (DAA) (*n* = 30) vs. non-dilated ascending aorta (NDAA) (*n* = 20). Subjects were also divided into four groups based on aortic valve morphology and aorta diameter: 16 patients had a dilated aorta and bicuspid aortic valve (DAA-BAV); 14 had a dilated aorta and tricuspid aortic valve (DAA-TAV); 15 had a non-dilated aorta and bicuspid aortic valve (NDAA-BAV); and 5 had a non-dilated aorta and tricuspid valve (NDAA-TAV).

**TABLE 1:** Patient’s demographics and characteristics according to aortic size and in reference group.

	DAA	NDAA	CG	*P*
	*N* = 30	*N* = 20	*N* = 40	
Age (years)	48.5 (37.8–57.3)	46 (40.5–48.5)	39.5 (32.3–45.5)	<0.05
Sex (female)	3 (10)	5 (25)	19 (47.5)	<0.01
Body mass index	28.8 (25.8–30.9)	28.9 (24.4–31.0)	24.9 (23.9–27.1)	<0.001
Arterial hypertension	13 (43.3)	4 (20)	0 (0)	<0.001
Ischemic heart diseases	2 (6.7)	0 (0)	0 (0)	NS
Diabetes mellitus	1 (3.3)	1 (5)	0 (0)	NS
Oncology	1 (3.3)	2 (10)	0 (0)	NS
Dyslipidemia	8 (26.7)	2 (10)	0 (0)	<0.001
Creatinine (μmol/l)	85 (75–96)	81.5 (70.5–97.5)	79.5 (65.5–93.8)	NS
CRP (mg/l)	1.75 (0.78–2.4)	1.2 (0.65–2.08)	0.75 (0.3–2.05)	NS
Leukocytes 10*9/l	7.11 (6.2–8.05)	6.4 (5.16–7.38)	6.71 (5.44–7.61)	NS
Aortic anulus (mm)	27.5 (25.0–30.0)	26 (23.0–30.0)	20 (0–21.8)	<0.001
Aortic sinus (mm)	50 (43.8–57.3)	40 (34.5–43.0)	33.5 (30.0–35.0)	<0.001
Ascending aorta (mm)	51 (46.8–57.0)	39.5 (35.8–44.5)	30 (27.3–33.8)	<0.001
Bicuspid aortic valve	16 (53.3)	15 (75)	0 (0)	<0.001
Stenosis	2 (6.7)	4 (20)	0 (0)	<0.01
Insufficiency	12 (40)	9 (45)	0 (0)	<0.001
Both	1 (3.3)	0 (0)	0 (0)	NS
Tricuspid aortic valve	14 (46.7)	5 (15)	40 (100)	<0.001
Stenosis	0 (0)	1 (5)	0 (0)	NS
Insufficiency	13 (43.3)	2 (10)	2 (5)	<0.001
Both	0 (0)	0 (0)	0 (0)	

CG, control group; DAA, dilated ascending aorta; NDAA, non-dilated ascending. Data are expressed as median (IQR) or number (%).

A control group (CG) (*n* = 40) was drawn from the local population and UHHK staff volunteers. Blood samples were collected from the 50 patients prior to surgery as well as from our control group. The study excluded subjects with congenital aortopathy, aortic dilatation syndromes, and endocarditis. The study was approved by the Institutional Review Board on 11 April 2017 (201706 S13P). Prior to surgery, each patient signed an informed consent form.

### Histological analysis

Full aortic samples were collected from the resected aortic walls of 30 patients. After excision, at least six segments taken across the circumference of the aorta were fixed in formalin, decalcified if necessary, embedded in paraffin, and sectioned. The morphology of the aortic wall was evaluated using hematoxylin-eosin (HE) and Elastica van Gieson (EVG) staining. A pathologist examined the samples and graded them semiquantitatively. The grading system of the samples was based on the consensus statement on surgical pathology of the aorta (10).

Elastic fiber fragmentation and loss (EFF/L) grade 0–III, mucoid extracellular matrix accumulation (MEMA) grade 0–III, smooth muscle cell nuclei loss/laminar medial collapse (SMCNL/LMC) grade 0–III, and medial fibrosis (MF) grade 0–III are the terms used in the histological description with grading. Based on the estimated area of involvement, this grading was subjective and on a semiquantitative scale. Grade 0 denotes no involvement, grade I denotes low involvement, grade II denotes middle involvement, and grade III denotes high involvement.

The pathologist identified all histopathological units. Aortic samples revealed medial degeneration (EFF/L + MEMA + SMCNL/LMC + MF), atherosclerosis, IgG aortitis, and normal aorta.

### TGF-β1 quantitative detection

Serum TGF-β1 levels were obtained with human quantitative sandwich enzyme immunoassay technique kit TGF-β1 ELISA kit (R&D Systems, Minneapolis, United States).

Blood was collected in 4 ml serum-separating tubes (Becton Dickinson), with a clotting time of 30 min at room temperature. Tubes with clotted blood were spanned for 15 min at 1,000 × g, causing serum to separate to the upper part of the tube. Separated serum was then aliquoted and stored at −70°C. Serum samples, all controls and calibrators were tested in duplicates, using human TGF-β1 ELISA kit according to the manufacturer’s instructions. Standard methodical workflow of measuring TGF-β1 concentration include acid activation and neutralization, subsequent incubation and washing, in order to obtain a concentration of active TGF-β1. At the end, absorbance of the samples and controls in the microplate was measured by a reader set to 450 nm as a primary wavelength (Elisa Reader Power Wave XS BIOTEK, Germany) (6, 14, 15).

This assay uses the principle of quantitative sandwich enzyme immunoassay technique. A monoclonal antibody specific for TGF-β1 is immobilized on the surface of the microplate wells. Standards, controls and patient’s samples in duplicates are pipetted into the wells and all of the TGF-β1 present in a sample bind with the immobilized antibody on the surface of the well. After washing away the unbound substances, an enzyme-linked polyclonal antibody specific for TGF-β1 is added to the wells. Antibody with enzyme links in a sandwich-like structure with TGF-β1 is immobilized during the first incubation. Another wash follows, in order to remove any unbound reagents. After washing, a substrate solution is added to the wells and the color that starts to appear correlates with the amount of TGF-β1 bound in the initial step. Finally, the color development is stopped and the intensity of the color is measured by photometry. In the analysis of samples, the latent form (LAP) of TGF-β1 was activated. The TGF-β1 concentrations in our experiment include both the active and latent forms.

### Statistical analysis

The data obtained are shown as either categorical variables (shown as frequency and percentage) or continuous variables [expressed as 50th (25th–75th) percentile]. Statistical differences in quantitative demographic and clinical characteristics between the three groups (DAA vs. NDAA vs. RG) of patients were performed using Kruskal-Wallis analysis of variance, in case of significance Dunn’s test and Bonferroni modified test of significance level were performed. Comparison of qualitative demographic and clinical characteristics was done using Fisher’s exact test and χ^2^. Spearman’s rank correlation coefficient was used to identify the association between histological abnormalities and TGF-β1, in addition aortic diameter and age were also correlated. The receiver operating characteristic (ROC) curve was determined and the area under the curve (AUC) was constructed to assess the areas under the curve (AUC) and the 95% confidence interval.

The statistical analyses were done with Statistical Software (2021). NCSS, LLC. Kaysville, Utah, United States, ncss.com/software/ncss. The differences were considered statistically significant with α < 0.05.

## Results

Table 1 summarizes the demographic and clinical characteristics of the participants in the study. Except for a higher incidence of arterial hypertension and dyslipidemia in the DAA group (*P* < 0.001), we found no significant differences in demographic parameters between the DAA and NDAA groups. In terms of clinical parameters, we discovered significant differences between DAA and NDAA in the frequency of bicuspid aortic valve. The control group (*n* = 40) included 19 females and 21 males with a median age of 39.5 years, none of whom had aortic valve disease or ascending aorta dilatation.

**FIGURE 1:**
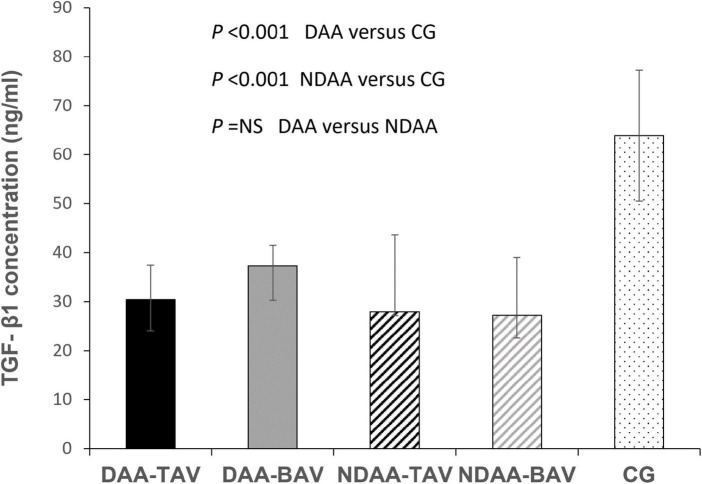
TGF-β1 concentrations in patients with dilated and non-dilated aortas according to aortic valve cuspidity. CG, control group; DAA-TAV, dilated ascending aorta- tricuspid aortic valve; DAA-BAV, dilated ascending aorta- bicuspid aortic valve; NDAA-TAV, non-dilated ascending aorta-tricuspid aortic valve; NDAA-BAV, non-dilated ascending aorta-bicuspid aortic valve.

TGF-β1 values in the three groups are shown in Table 2 (DAA 32.5 (28.37–40.03) ng/ml, NDAA 27.68 (25.46–38.24) ng/ml, and reference group 63.92 (50.49–77.22) ng/ml). We discovered a significant 2-fold decrease in TGF-β1 levels in the blood samples of patients with aortic dilatation compared to the reference group (32.5 vs. 63.92; *P* < 0.001), as well as a significant 2.3-fold decrease in the patients with non-dilated aorta (27.68 vs. 63.92; *P* < 0.001). There was no significant difference in TGF-β1 levels between the DAA and NDAA groups (32.5 vs. 27.68; *P* < 0.365) (Figure 1). When patients were grouped according to aortic valve phenotype, BAV and TAV group, TGF-β1 concentrations were found again to be significantly different between BAV group and control group (*P* < 0.001) and TAV group and control group (*P* < 0.001). No significant difference was found between patients with BAV and TAV independent of dilatation.

**TABLE 2:** TGF-β1 concentrations in groups according to aortic size and in reference group.

	DAA	NDAA	CG	*P*
	*N* = 30	*N* = 20	*N* = 40	
TGF-β1 (ng/ml)	32.50 (28.37–40.03)	27.68 (25.46–38.24)	63.92 (50.49–77.22)	<0.001

CG, control group; DAA, dilated ascending aorta; NDAA, non-dilated ascending.

Data are expressed as median (IQR).

*P* < 0.001 DAA vs. CG.

*P* < 0.001 NDAA vs. CG.

*P* = NS DAA vs. NDAA.

When patients were divided into groups based on aortic valve phenotype, there was no statistically significant difference in TGF-β1 levels between DAA-TAV and DAA-BAV [30.39 (24.1–37.44) vs. 37.35 (30.27–41.5); *P* = 0.19], nor between NDAA-TAV and NDAA-BAV [27.94 (27.19–43.66) vs. 27.22 (22.57–39.03); *P* = 0.337].

When comparing the patients with bicuspid aortic valve, there was also no significant difference between DAA-BAV and NDAA-BAV [37.35 (30.27–41.5) vs. 27.22 (22.57–39.03); *P* = 0.26].

The correlation between TGF-β1 levels and age or ascending aorta diameter, as well as anulus and sinus size, was found to be poor for all the variables studied (Table 3). There was no significant relationship between TGF-β1 levels and the grade of ascending aorta histopathological abnormalities (Table 4).

**TABLE 3:** Spearman’s correlation analyses for TGF-β1 concentration vs. age and ascending aorta, aortic sinus, and annulus size according to aortic dilatation.

	DAA + NDAA		DAA		NDAA		CG	
	*N* = 50	Spearman’s *P*-value	*N* = 30	Spearman’s *P*-value	*N* = 20	Spearman’s *P*-value	*N* = 40	Spearman’s *P*-value
Age	−0.0737	0.653	−0.194	0.328	0.0849	0.653	0.227	0.244
Aortic anulus	−0.107	0.443	−0.0159	0.674	−0.106	0.374	−0.0634	0.309
Aortic sinus	−0.0111	0.934	−0.101	0.782	−0.111	0.673	0.261	0.101
Ascending aorta	0.218	0.484	−0.169	0.933	0.126	0.36	0.383	0.0738

CG, control group; DAA, dilated ascending aorta; NDAA, non-dilated ascending.

**TABLE 4:** Patient distribution by histological score according to the TGF-β1 concentration.

	N	TGF-β 1	*P-value*
EFF/L			0.593
0. No involvement	16	30.7 (29.25–40.05)	
1. Low	0	*None*	
2. Middle	2	29.16 (13.6–44.72	
3. High	12	31.29 (27.49–39.11)	
MEMA			0.603
0. No involvement	11	36.67 (28.86–41.98)	
1. Low	4	24.06 (14.5–41.27)	
2. Middle	1	40.03	
3. High	14	31.36 (28.67–38.13)	
SMCNL/LMC			0.705
0. No involvement	21	32.27 (26.09–42)	
1. Low	2	34.01 (30.91–37.11)	
2. Middle	1	40.03	
3. High	6	31.71 (25.86–38.13)	
MF			0.887
0. No involvement	19	36.67 (26.9–41.98)	
1. Low	2	36.47 (30.91–42.03)	
2. Middle	4	27.76 (15.25–32.45)	
3. High	5	32.74 (29.66–38.68)	

EFF/L, Elastic fiber fragmentation and loss; MEMA, Mucoid extra cellular matrix accumulation; MF, Medial fibrosis; SMCNL/LMC, Smooth muscle cell nuclei loss/laminar medial collapse. Data are expressed as median (IQR).

**FIGURE 2:**
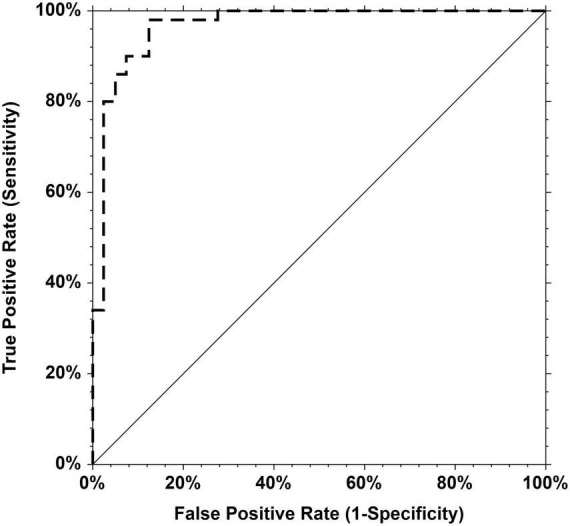
Receiver operating characteristic (ROC) curves for TGF-β1. Area under the curve = 0.9670 for predicting aortic valvulopathy-aorthopathy.

The area under the receiver operating curve (AUC-ROC) for the serum TGF-β1 concentration was 0.967 (Standard Error 0.018, 95% CI 0.901–0.989) for predicting aortic valvulopathy-aorthopathy. The cut-off point was 48.72 ng/mL with the sensitivity of 0.980 and the specificity of 0.875 (Figure 2).

## Discussion

The European guidelines on the diagnosis and treatment of aortic disease recommend resection of the aortic root or ascending aorta if diameter exceeds 45 mm, when scheduling aortic valve surgery (16). In contrast, this procedure is recommended in BAV patients with risk factors, with no need for valve surgery, when the aortic diameter is ≥50 mm. In all patients surgery is recommended in diameter ≥55 mm. The clinical reasoning for this approach to aortopathy is primarily based on autopsy studies (17). The development of plasma biomarkers with sufficient diagnostic accuracy as a screening tool for patients with ascending aorta dilatation is critical because they are typically asymptomatic during the clinical course of the disease and are frequently diagnosed as a result of potentially fatal complications. So far, no biomarker has proven to be adequate (18, 19).

Several potential biomarkers have been studied. The role of inflammation in the development of aortic aneurysm is becoming evident, along with a potential diagnostic marker for this correlation. Chemokines released by immune cells seem to play an important role in medial wall degeneration in thoracic aorta aneurysm. Cytokines, such as IL- 1β, IL-6, IFN-γ, or TNF-α have been reported to have higher concentrations in the peripheral blood of patients with aortic aneurysm (20–22). Elevated plasma d-dimers and hypersensitive CRP have been studied in patients with aortic dissection, but their roles in screening for thoracic aneurysm are yet unknown (23). There is currently no inflammatory biomarker or combination of biomarkers that has the diagnostic accuracy to detect aortic aortopathy and the development of aortic aneurysm. Circulating microRNAs have gained popularity as potential biomarkers of many pathological conditions, as well as aortic aneurysm. The role of microRNAs in the regulation of aneurysm formation has been demonstrated. However, there has been no clinical impact as of yet (24, 25). Plasma metalloproteinase, advanced glycation end-products levels and other potential molecules have been recently investigated (18). So far, none of the previously reported substances can be used in clinical practice to detect aortic aneurysm formation or predict future development.

Several studies have found that the TGF-β1 signaling pathway is important in the development of abdominal and thoracic aortic aneurysms (2, 18, 19, 26, 27). These findings in patients with connective tissue disorders have been published (28). Hilebrand et al. reported elevated total TGF-β1 levels in the entire spectrum of genetic aortic syndromes (27). Later, Matt et al. authors reported significant changes in circulating TGF-β1 in Marfan syndrome patients compared with control individuals (29).

In our study, we looked at blood plasma TGF-β1 levels in patients with non-syndromic aortic dilation with tricuspid or bicuspid aortic valves and normal or dilated ascending aortas, as well as in a control group. In the current study we found that plasma levels of TGF-β1 differ significantly between patients with dilated aorta or aortic valve disease (valvulopathy) and a healthy population. In contrast, no significant difference was found between levels of TGF-β1 according to aortic valve phenotype (BAV vs. TAV patients). Similar changes in serum TGF-β1 concentration were reported by Forte et al. in the study of BAV associated aortopathy (18). They observed a decrease in plasma levels in BAV patients compared to the control group. Moreover, TGF-β1/endoglin ratio correlated with the progression of the aortic dilatation in patients with stenotic BAV, and suggest that it could be a marker for risk stratification.

Sepetiene et al. reported a significant association between TGF-β1 concentration and a dilatative pathology of the ascending aorta (DPAA), but the mechanism of cause and effect was not determined (6). However, patients with acute aortic dissection were included in their study, compared to our study, and TGF-β1 concentrations in this subgroup differed the most from the control group. In contrast to the Rueda-Martínez et al. study we found a significant 2-fold decrease in the levels of TGF-β1 in the blood of patients with aortic dilatation compared to the reference group (28). Although we found significantly different values of TGF-β1 between DAA and NDAA compared to the reference group, we did not find a correlation between concentrations of TGF-β1 and ascending aorta dilatation size. Our findings were similar for the correlation of TGF-β1 levels and the grade of various histopathological units.

An important finding from the literature is the differing TGF-β1 concentration levels found among studies. It should be noted that in our study we measured a decreased level of TGF-β1 plasma concentration in the disease group compared to the reference group due to the process of activation. In the analysis of the samples, the latent form (LAP) of TGF-β1 was also activated. The total TGF-β1 concentrations included both the active and latent forms of TGF-β1 in our experiment, which explains the difference compared to the results of Sapetiene et al. (6) and Rueda-Martínez et al. (30), but are consistent to study published by Forte et al. (18). Our results, in comparison to previous studies, are controversial as our data show lower levels of TGF-β1 in patients with diseased aorta. We hypothesize that this could be due to our method of measurement of both the active and latent form of TGF-β1, the latter we suspect is used up during the reparative or inflammation process in the tissue of a diseased aorta. TGF-β1 concentration variation between the studies illustrates the importance of the testing methods used and should always be considered when comparing results from different studies.

Similar concentrations in DAA and NDAA patients can be attributed to the aortic valve and ascending aorta developing from neural crest cells during embryonic development (31–34). TGF-β1 levels were lower in the coronary artery ectasia group than in the control group, according to Ser et al. (35). This information provided us with additional information indicating possible similarities in the ascending aorta’s anatomical structures.

Our findings as well as the literature suggest that TGF-β1 concentrations are significantly changed not only in patients with syndromic thoracic aortic aneurysm but also in non-syndromic aortic dilatation, as well as in patients with aortic valve pathology. TGF-β1 could not be used as a direct diagnostic marker of isolated aortic wall dilatation, at least in this series. TGF-β1 plasma levels did not correlate with the severity of TAA dimension or histopathological wall changes.

### Limitations

The primary limitation of this study is the small sample size. Furthermore, some analytic techniques were not used in this study, specifically genetic screening, immunohistochemistry, and quantitative fluorescence microscopy. Future research with TGF-β1 measurement is required to validate our findings and test our hypothesis.

## Conclusion

The current study’s data provide additional evidence that plasma levels of TGF-β1 are significantly different in non-syndromic patients with aortic dilatation compared to a control group. Significant differences of TGF-β1 plasma concentrations are also related to severe aortic valvulopathy, without the need for proven aortic dilatation. TGF-β1 concentration had no relationship with histopathological changes nor aortic diameter. The mechanism of cause and effect has yet to be determined. TGF-β1 is sensitive to changes in the aortic wall and valve, but it is not specific.

## Data availability statement

The raw data supporting the conclusions of this article will be made available by the authors, without undue reservation.

## Ethics statement

The studies involving human participants were reviewed and approved by the Institutional Review Board, University Hospital Hradec Králové. The patients/participants provided their written informed consent to participate in this study.

## Author contributions

MK: substantial contribution to the concept, drafting of the article, data collection, and data analysis. MP: substantial contribution to the concept, drafting of the article, and critical revision of the manuscript. LZ: samples analysis and revision of the manuscript. VS: histological examination and figures. ST and PB: data collection and drafting of the manuscript. JV: critical revision of the article and approval of the article. All authors contributed to the article and approved the submitted version.

## References

[1] Hiratzka LF Bakris GL Beckman JA Bersin RM Carr VF Casey DE Jr. 2010 ACCF/AHA/AATS/ACR/ASA/SCA/SCAI/SIR/STS/SVM guidelines for the diagnosis and management of patients with thoracic aortic disease: a report of the American college of cardiology foundation/American heart association task force on practice guidelines, American association for thoracic surgery, American college of radiology, American stroke association, society of cardiovascular anesthesiologists, society for cardiovascular angiography and interventions, society of interventional radiology, society of thoracic surgeons, and society for vascular medicine. *Circulation.* (2010) 121:e266–369. 20233780 10.1161/CIR.0b013e3181d4739e

[2] Gillis E Van Laer L Loeys BL. Genetics of thoracic aortic aneurysm: at the crossroad of transforming growth factor-beta signaling and vascular smooth muscle cell contractility. *Circ Res.* (2013) 113:327–40. 10.1161/CIRCRESAHA.113.300675 23868829

[3] Losenno KL Goodman RL Chu MW. Bicuspid aortic valve disease and ascending aortic aneurysms: gaps in knowledge. *Cardiol Res Pract.* (2012) 2012:145202. 23198270 10.1155/2012/145202 PMC3503270

[4] Prakash SK Bossé Y Muehlschlegel JD Michelena HI Limongelli G Della Corte A A roadmap to investigate the genetic basis of bicuspid aortic valve and its complications: insights from the international BAVCon (bicuspid aortic valve consortium). *J Am Coll Cardiol.* (2014) 64:832–9. 10.1016/j.jacc.2014.04.073 25145529 PMC4485610

[5] Verma S Siu SC. Aortic dilatation in patients with bicuspid aortic valve. *N Engl J Med.* (2014) 370:1920–9. 24827036 10.1056/NEJMra1207059

[6] Sepetiene R Patamsyte V Zukovas G Jariene G Stanioniene Z Benetis R Blood plasma TGF- beta1 concentration in sporadic dilatative pathology of ascending aorta: more questions than answers. *PLoS One.* (2015) 10:e0129353. 10.1371/journal.pone.0129353 26103587 PMC4478017

[7] Annes JP Munger JS Rifkin DB. Making sense of latent TGFbeta activation. *J Cell Sci.* (2003) 116:217–24. 10.1242/jcs.00229 12482908

[8] Lindsay ME Dietz HC. Lessons on the pathogenesis of aneurysm from heritable conditions. *Nature.* (2011) 473:308–16. 10.1038/nature10145 21593863 PMC3622871

[9] Jain D Dietz HC Oswald GL Maleszewski JJ Halushka MK. Causes and histopathology of ascending aortic disease in children and young adults. *Cardiovasc Pathol.* (2011) 20:15–25. 10.1016/j.carpath.2009.09.008 19926309 PMC3046386

[10] Halushka MK Angelini A Bartoloni G Basso C Batoroeva L Bruneval P Consensus statement on surgical pathology of the aorta from the society for cardiovascular pathology and the association for European cardiovascular pathology: II. Noninflammatory degenerative diseases – nomenclature and diagnostic criteria. *Cardiovasc Pathol.* (2016) 25:247–57. 10.1016/j.carpath.2016.03.002 27031798

[11] He R Guo DC Estrera AL Safi HJ Huynh TT Yin Z Characterization of the inflammatory and apoptotic cells in the aortas of patients with ascending thoracic aortic aneurysms and dissections. *J Thorac Cardiovasc Surg.* (2006) 131:671–8. 10.1016/j.jtcvs.2005.09.018 16515922

[12] Schmid FX Bielenberg K Schneider A Haussler A Keyser A Birnbaum D. Ascending aortic aneurysm associated with bicuspid and tricuspid aortic valve: involvement and clinical relevance of smooth muscle cell apoptosis and expression of cell death-initiating proteins. *Eur J Cardiothorac Surg.* (2003) 23:537–43. 10.1016/s1010-7940(02)00833-3 12694773

[13] Wortmann M Skorubskaya E Peters AS Hakimi M Böckler D Dihlmann S. Necrotic cell debris induces a NF-kappaB-driven inflammasome response in vascular smooth muscle cells derived from abdominal aortic aneurysms (AAA-SMC). *Biochem Biophys Res Commun.* (2019) 511:343–9. 10.1016/j.bbrc.2019.02.051 30782482

[14] Munger JS Harpel JG Giancotti FG Rifkin DB. Interactions between growth factors and integrins: latent forms of transforming growth factor-beta are ligands for the integrin alphavbeta1. *Mol Biol Cell.* (1998) 9:2627–38. 10.1091/mbc.9.9.2627 9725916 PMC25536

[15] Munger JS Harpel JG Gleizes PE Mazzieri R Nunes I Rifkin DB Latent transforming growth factor-beta: structural features and mechanisms of activation. *Kidney Int.* (1997) 51:1376–82. 9150447 10.1038/ki.1997.188

[16] Vahanian A Beyersdorf F Praz F Milojevic M Baldus S Bauersachs J 2021 ESC/EACTS guidelines for the management of valvular heart disease. *Eur Heart J.* (2022) 43:561–632. 34453165 10.1093/eurheartj/ehab395

[17] Larson EW Edwards WD. Risk factors for aortic dissection: a necropsy study of 161 cases. *Am J Cardiol.* (1984) 53:849–55. 10.1016/0002-9149(84)90418-1 6702637

[18] Forte A Bancone C Cobellis G Buonocore M Santarpino G Fischlein TJM A possible early biomarker for bicuspid aortopathy: circulating transforming growth factor beta-1 to soluble endoglin ratio. *Circ Res.* (2017) 120:1800–11. 10.1161/CIRCRESAHA.117.310833 28420669

[19] Gomez D Al Haj Zen A Borges LF Philippe M Gutierrez PS Jondeau G Syndromic and non-syndromic aneurysms of the human ascending aorta share activation of the Smad2 pathway. *J Pathol.* (2009) 218:131–42. 10.1002/path.2516 19224541

[20] Skotsimara G Antonopoulos A Oikonomou E Papastamos C Siasos G Tousoulis D. Aortic wall inflammation in the pathogenesis, diagnosis and treatment of aortic aneurysms. *Inflammation.* (2022) 45:965–76. 35076833 10.1007/s10753-022-01626-z

[21] Juvonen J Surcel HM Satta J Teppo AM Bloigu A Syrjälä H Elevated circulating levels of inflammatory cytokines in patients with abdominal aortic aneurysm. *Arterioscler Thromb Vasc Biol.* (1997) 17:2843–7. 9409264 10.1161/01.atv.17.11.2843

[22] Batra R Suh MK Carson JS Dale MA Meisinger TM Fitzgerald M IL-1β (Interleukin-1β) and TNF-α (tumor necrosis factor-α) impact abdominal aortic aneurysm formation by differential effects on macrophage polarization. *Arterioscler Thromb Vasc Biol* (2018) 38:457–63. 29217508 10.1161/ATVBAHA.117.310333 PMC7450719

[23] Yuan SH Shi YH Wang JJ Lü FQ Gao S. Elevated plasma D-dimer and hypersensitive C-reactive protein levels may indicate aortic disorders. *Rev Bras Cir Cardiovasc.* (2011) 26:573–81. 10.5935/1678-9741.20110047 22358272

[24] Ikonomidis JS Ivey CR Wheeler JB Akerman AW Rice A Patel RK Plasma biomarkers for distinguishing etiologic subtypes of thoracic aortic aneurysm disease. *J Thorac Cardiovasc Surg.* (2013) 145:1326–33. 10.1016/j.jtcvs.2012.12.027 23312977 PMC4057430

[25] Siasos G Bletsa E Stampouloglou PK Oikonomou E Tsigkou V Paschou SA MicroRNAs in cardiovascular disease. *Hellenic J Cardiol.* (2020) 61:165–73. 32305497 10.1016/j.hjc.2020.03.003

[26] Gomez D Kessler K Michel JB Vranckx R. Modifications of chromatin dynamics control Smad2 pathway activation in aneurysmal smooth muscle cells. *Circ Res.* (2013) 113:881–90. 10.1161/CIRCRESAHA.113.301989 23825360

[27] Hillebrand M Millot N Sheikhzadeh S Rybczynski M Gerth S Kölbel T Total serum transforming growth factor-beta1 is elevated in the entire spectrum of genetic aortic syndromes. *Clin Cardiol.* (2014) 37:672–9. 10.1002/clc.22320 25113270 PMC6649456

[28] Wheeler JB Ikonomidis JS Jones JA. Connective tissue disorders and cardiovascular complications: the indomitable role of transforming growth factor-β signaling. *Adv Exp Med Biol.* (2021) 1348:161–84. 34807419 10.1007/978-3-030-80614-9_7 PMC8905527

[29] Matt P Schoenhoff F Habashi J Holm T Van Erp C Loch D Circulating transforming growth factor-beta in Marfan syndrome. *Circulation.* (2009) 120:526–32. 19635970 10.1161/CIRCULATIONAHA.108.841981 PMC2779568

[30] Rueda-Martinez C Lamas O Carrasco-Chinchilla F Robledo-Carmona J Porras C. Increased blood levels of transforming growth factor beta in patients with aortic dilatation. *Interact Cardiovasc Thorac Surg.* (2017) 25:571–4. 28666329 10.1093/icvts/ivx153

[31] Ausoni S Sartore S. Cell lineages and tissue boundaries in cardiac arterial and venous poles: developmental patterns, animal models, and implications for congenital vascular diseases. *Arterioscler Thromb Vasc Biol.* (2001) 21:312–20. 10.1161/01.atv.21.3.312 11231908

[32] Kappetein AP Gittenberger-de Groot AC Zwinderman AH Rohmer J Poelmann RE Huysmans HA. The neural crest as a possible pathogenetic factor in coarctation of the aorta and bicuspid aortic valve. *J Thorac Cardiovasc Surg.* (1991) 102:830–6. 1960986

[33] Kirby ML Waldo KL. Role of neural crest in congenital heart disease. *Circulation.* (1990) 82:332–40. 2197017 10.1161/01.cir.82.2.332

[34] Morrison-Graham K Schatteman GC Bork T Bowen-Pope DF Weston JA. A PDGF receptor mutation in the mouse (Patch) perturbs the development of a non-neuronal subset of neural crest-derived cells. *Development.* (1992) 115:133–42. 10.1242/dev.115.1.133 1638976

[35] Ser OS Çetinkal G Kiliçarslan O Dalgıç Y Batit S Keskin K The comparison of serum TGF-beta levels and associated polymorphisms in patients with coronary artery ectasia and normal coronary artery. *Egypt Heart J.* (2021) 73:32. 10.1186/s43044-021-00153-w 33788038 PMC8012455

